# 

**Published:** 1856-12

**Authors:** 


VOL. V. NEW SERIES. No. 12.

THE

### NORTH-WESTERN MEDICAL AND SURGICAL JOURNAL.

EDITED BY

N. S. DAVIS, M.D.,

PROFESSOR OF PRINCIPLES AND PRACTICE OF MEDICINE AND CLINICAL MEDICINE IN RUSH MEDICAL COLLEGE; ONE OF THE PHYSICIANS TO THE MERCY HOSPITAL; FELLOW OF THE COLLEGE OF PHYSICIANS AND SURGEONS, NEW YORK; MEMBER OF THE MEDICAL SOCIETY OF THE STATE OF NEW YORK, AND OF THE STATE OF ILLINOIS; MEMBER OF THE AMERICAN MEDICAL ASSOCIATION, &C. &C.

PUBLISHED MONTHLY.

AT TWO DOLLARS PER ANNUM, IN ADVANCE.

**DECEMBER, 1856.**

CHICAGO:
ROBERT FERGUS, PRINTER
189 LAKE STREET.

VOL. XIII. WHOLE SERIES. No. 12.

